# Graph ranking for exploratory gene data analysis

**DOI:** 10.1186/1471-2105-10-S11-S19

**Published:** 2009-10-08

**Authors:** Cuilan Gao, Xin Dang, Yixin Chen, Dawn Wilkins

**Affiliations:** 1 Department of Mathematics, The University of Mississippi, University, MS, 38677, USA; 2 Computer & Information Science Department, The University of Mississippi, University, MS, 38677, USA

## Abstract

**Background:**

Microarray technology has made it possible to simultaneously monitor the expression levels of thousands of genes in a single experiment. However, the large number of genes greatly increases the challenges of analyzing, comprehending and interpreting the resulting mass of data. Selecting a subset of important genes is inevitable to address the challenge. Gene selection has been investigated extensively over the last decade. Most selection procedures, however, are not sufficient for accurate inference of underlying biology, because biological significance does not necessarily have to be statistically significant. Additional biological knowledge needs to be integrated into the gene selection procedure.

**Results:**

We propose a general framework for gene ranking. We construct a bipartite graph from the Gene Ontology (GO) and gene expression data. The graph describes the relationship between genes and their associated molecular functions. Under a species condition, edge weights of the graph are assigned to be gene expression level. Such a graph provides a mathematical means to represent both species-independent and species-dependent biological information. We also develop a new ranking algorithm to analyze the weighted graph via a kernelized spatial depth (KSD) approach. Consequently, the importance of gene and molecular function can be simultaneously ranked by a real-valued measure, KSD, which incorporates the global and local structure of the graph. Over-expressed and under-regulated genes also can be separately ranked.

**Conclusion:**

The gene-function bigraph integrates molecular function annotations into gene expression data. The relevance of genes is described in the graph (through a common function). The proposed method provides an exploratory framework for gene data analysis.

## Background

### Introduction

Microarray technology has made it possible to simultaneously monitor the expression levels of thousands of genes during important biological processes and across collections of related samples. Elucidating the patterns hidden in gene expression data offers a tremendous opportunity for an enhanced understanding of functional genomics. However, the large number of genes greatly increases the challenges of analyzing, comprehending and interpreting the resulting mass of data. Selecting a subset of important genes is necessary to address the challenge for two primary reasons. First, multivariate methods are prone to overfitting. This problem is aggravated when the number of variables is large compared to the number of examples, and even worse for gene expression data which usually has ten or twenty thousand genes but with only a very limited number of samples. It is not uncommon to use a variable ranking method to filter out the least promising variables before using a multivariate method. The second reason for ranking the importance of genes is that identifying important genes is, in and of itself, interesting. For example, to answer the question of what genes are important for distinguishing between cancerous and normal tissue may lead to new medical practices.

Gene selection has been investigated extensively over the last decade by researchers from the statistics, data mining and bioinformatics communities. There are basically two approaches. One approach treats gene selection as a pre-processing step. It usually comes with a measure to rank genes. Fold change is a simple measure used in [1]. Dudoit, *et al*. [2] performed a selection of genes based on the between-group and within-group variance ratios. Golub, *et al*. [3] used a different method for standardizing the data for selecting genes. Pepe, *et al*. [4] considered two measures related to the Receiver Operating Characteristic curve (ROC) for ranking genes. Strength of statistical evidence, such as p-values of hypothesis testing [5], are also commonly used measures for gene selection. Storey and Tibshirani [6] proposed a measure of significance called q-value based on the concept of false discovery rate. The other common approach to gene selection embeds gene selection into a specific learning procedure. Fan and Li [7] proposed penalized likelihood methods for regression to select variables and estimate coefficients simultaneously. Lee, *et al*. [8] proposed a hierarchical Bayesian model for gene selection. They employed latent variables to specialize the model to a regression setting and used a Bayesian mixture prior to perform the variable selection. Recursive feature elimination (RFE) methods with support vector machines (SVM), e.g. [9-12], have been shown to be successful for gene selection and classification. *L*_1 _SVMs perform variable selection automatically by solving a quadratic optimization problem, e.g. [13-15]. Diáz, *et al*. [16] applied a random forest algorithm for classification and at the same time for selecting genes based on the permuted importance score. Mukherjee and Roberts [17] provided a theoretical analysis of gene selection, in which the probability of successfully selecting relevant genes, using a given gene ranking function, is explicitly calculated in terms of population parameters. For a more comprehensive survey of this subject, the reader is referred to [18,19], and [20].

In most of the cases, genes selected by the aforementioned procedures are not sufficient for accurate inference of the underlying biology, because biological significance does not necessarily have to be statistically significant [21]. For example, suppose the gene with low differential expression is a transcription factor that controls the expression of some other genes. The transcription factor itself may be activated by the treatment but its expression may not be significantly changed. Hence, an ideal selection procedure should be able to highlight the transcription factor. To do so, additional biological knowledge must be integrated into it. With the development of biological knowledge databases, biologically interesting sets of genes, for example genes that belong to a pathway or genes known to have the same molecular function, can be compiled, for example from Gene Ontology [22], see GO Consortium (2008). There have been many publications combining gene expression with GO lately. One common approach is to find enriched gene sets annotated by GO terms which are over-represented among the differentially expressed genes in the analysis of microarray data. See [23-26], and [27] for details of enrichment. The other approach is to use a GO graph to improve identification of differentially expressed genes. Morrison, *et al*. [28] constructed a gene-gene graph derived from GO and used GeneRank, which is a modification of PageRank (the ranking algorithm used in Google search engine), for prioritizing the importance of genes. Gene expression data was cleverly used to specify "the personalization vector" in PageRank. Ma *et al*. [29] first computed an individual score for each gene from gene expression profiles, then combined the scores of a gene and its direct and indirect neighbors in the gene-gene graph derived from GO or protein-protein interaction network to obtain a more accurate gene ranking. Daigle and Altman [30] developed a probabilistic model that integrates biological knowledge with microarray data to identify differentially expressed (DE) genes. They introduced a latent binary variable (DE/not DE) and used a learning algorithm on a stochastic, binary state network to estimate ranking score. Srivastava, *et al*. [31] used the GO structure to compute the similarity between genes and combined gene expression data in a ridge regression for gene selection. Clearly, an approach integrating GO and gene data captures dependent structure of genes without sacrificing gene-level resolution. It provides more reliable results than the methods relying on gene expression data alone, which is justified later.

In this paper, we propose an exploratory framework of gene ranking that utilizes gene expression profiles and GO annotations. The contributions of this paper are described as follows.

### Our contributions

• *Bi-graph representation of biological information of genes*. We extract biological information from the GO database. One of the three GO ontologies (molecular function) is used (the other two types of annotations biological process and cellular component can be used similarly). A bipartite graph is constructed with one partition being genes and the other molecular functions. If a gene is associated with a particular function, the gene and the function are joined by an edge. Such a graph structure represents species-independent biological knowledge among genes indirectly (through common functions). Furthermore, using gene expression studies, the weight of the edge is assigned to be the expression level of the gene associated with the edge. This integrates the species-dependent information into the graph. The weighted graph conveys gene dependency structure nicely.

• *A new graph ranking algorithm*. We introduce a new measure, *kernelized spatial depth (KSD)*, to rank the nodes of a graph. Spatial depth (SD) provides a center-outward ordering of a data set in an Euclidean space ℝ^*d*^. It is a global concept. KSD generalizes the notion of spatial depth by incorporating the local perspective of the data set. Applying KSD to a graph provides the ranking of nodes, which takes into consideration both global and local structures of the graph. For sparse graphs, the algorithm is efficient with computational complexity (*n*^2^), where *n *is the number of nodes of the graph. The algorithm can be easily modified to handle dynamic data sets. It can also be parallelized to scale up for large data sets.

• *Better interpretation*. Under a specified condition, not only is the importance of genes ranked, but the importance of functions is also ranked. This provides us with a better understanding and insight into the roles of various genes and molecular functions by analyzing bigraphs with gene expression profiles under different conditions. We demonstrate the performance of the proposed procedure using gene data from Gene Expression Omnibus (GEO). The new methods exhibit a higher level of biological relevance than competing methods.

Unlike a gene-gene network construction used in GeneRank, the gene-function bigraph structure has several advantages. It combines the gene expression profiles easily and naturally by assigning them to be weights of the graph. In addition, the importance of genes and molecular functions can be simultaneously ranked. Bipartite graph modeling was also used by Dhillon [32] and Zha, *et al*. [33] to co-cluster documents and words due to those advantages. Tanay, *et al*. [34] formed a gene-condition bigraph to find gene clusters in gene expression data.

The rest of this paper is organized as follows. After a brief introduction of some preliminaries on graphs, we introduce the KSD measure to rank vertices of a graph, followed by a discussion of choice of kernels and their comparison. In application, gene-function bigraphs are constructed to combine biological species-independent knowledge extracted from GO and species-dependent information contained in gene expression profiles. We apply our KSD ranking method to real data sets. Our conclusions and discussion are given in the last section.

## Methods

### Preliminaries of graphs and a motivating example

A graph *G *consists of a set of vertices (nodes) *V *and a set of edges *E *that connect vertices. The vertices are entities of interest and the edges represent relationships between the entities. Edges can be assigned positive weights *W *to quantify how strong the relationships are. Such a graph is called a weighted graph. Un-weighted graphs are just the special case with all the weights equally being 1.

A *bipartite *graph (or bigraph) is a graph whose vertices can be divided into two disjoint sets *V*_1 _and *V*_2 _such that every edge connects a vertex in *V*_1 _to one in *V*_2_. In our application, a bipartite graph is constructed with one set of vertices being genes and the other set of vertices being one of the Gene Ontology (GO) molecular functions.

The *degree *of a vertex *v *∈ *V *denoted as *d*_*v *_is defined as the sum of the weights related to *v*, i.e. *d*_*v *_= Σ_*u*_*W *(*v*, *u*); (*v*, *u*) ∈ *E*. Obviously, for an un-weighted graph, the degree of *v *is the number of incident edges.

**Figure 1:**
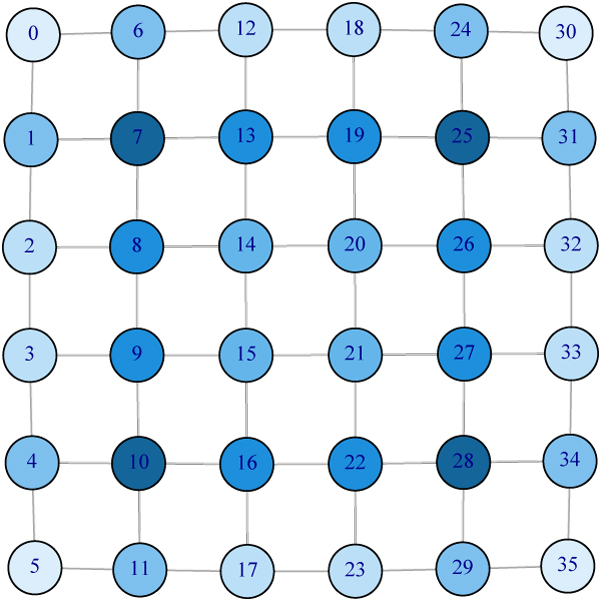
**Illustration of ranking by PageRank on a 6 × 6 grid**. Rankings by degree and by PageRank fail in this simple example. Vertices on the inner two layers have the same degree, 4. But the difference of those vertices can be measured by KSD. The colors from dark blue to light blue represent changes of the rank from high to low. PageRank with the damping parameter 1 yields same ordering as ranking by degree. The plot is the ordering result by PageRank with the damping parameter ∈ (0, 1).

**Figure 2:**
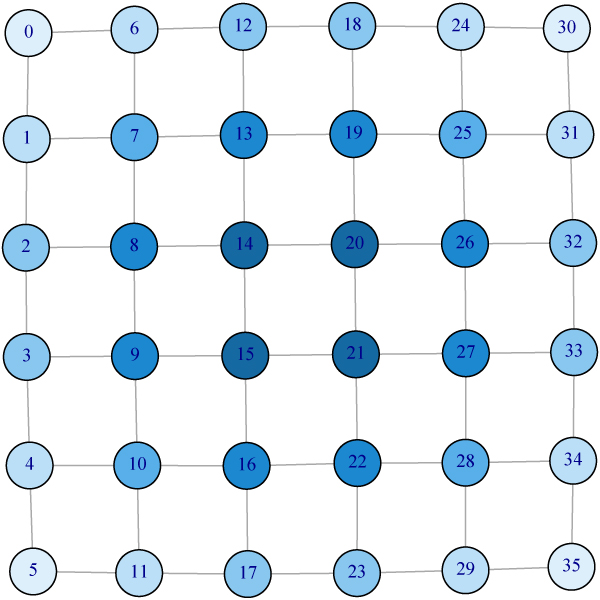
**Illustration of ranking by KSD on a 6 × 6 grid**. Ordering induced by KSD agrees well with what we expect. It demonstrates KSD is a promising measure for graph ranking.

Vertices with high degree play an important role in the graph. Ranking vertices purely by degree, however, may fail because the degree only contains the local information of a graph. For example, Figure 1 shows a 6 by 6 grid graph with 36 vertices. Vertices on the two inner layers have the same degree, four. There is no difference between Vertex 10 and Vertex 15 if one ranks them by their degrees. But intuitively Vertex 15 should be more "central" than Vertex 10 in the sense that it takes fewer steps to reach any vertex if one starts from Vertex 15 than it does from Vertex 10. PageRank with damping parameter 1 produces the same ordering as ranking by degree. For other values of damping parameter, the ranking of PageRank is provided by Figure 1. The colors from dark blue to light blue represent changes of the ranking score from large to small. Vertices 7, 10, 25 and 28 (the centers of four 3 by 3 grids) have the highest rank. Such ordering also demonstrates local focus. We propose kernelized spatial depth to rank vertices. The ordering induced by KSD (Fig 2) agrees well with what we expect. It suggests KSD as a promising measure for graph ranking.

### Spatial depth and kernelized spatial depth

We first introduce spatial depth in the Euclidean space ℝ^*d*^, then generalize it to kernelized spatial depth, which is the spatial depth on the feature space induced by a positive kernel. In order to extend the concept of KSD to a graph, the kernel on the graph must be specified. We define several graph kernels and present the KSD algorithm to obtain the depth of every vertex of the graph.

#### Spatial depth

Statistical data depth provides a center-outward ordering of a point in ℝ^*d *^with respect to a data set or a distribution. Multi-dimensional points can be ranked based on their depth. Among various notions of depth functions, spatial depth is appealing due to its computational ease and mathematical tractability [35]. The spatial depth of a point *x *∈ ℝ^*d *^with respect to a data set  = {*x*_1_, *x*_2_,...,*x*_*n*_} is defined as,(1)

From the definition, it is not difficult to see that points deep inside a data cloud receive high depth and those on the outskirts get lower depth. Each observation from a data set contributes equally, as a unit vector, to the value of the depth function. In this sense, spatial depth takes a global view of the data set. On the one hand, the spatial depth downplays the significance of distance and hence reduces the impact of those extreme observations whose extremity is measured in (Euclidean) distance, so that it gains resistance against these extreme observations. Robustness is a favorite property of spatial depth [36]. Ding, *et al*. [37] constructed a robust clustering algorithm based on it. On the other hand, the robustness of the depth function trades off some distance measurement, resulting in certain loss of the measurement of (dis)similarity of the data points. To overcome this limitation of spatial depth, Chen, *et al*. [38] proposed kernelized spatial depth (KSD) incorporating into the depth function a distance metric (or a similarity measure) induced by a positive definite kernel function.

#### Kernelized spatial depth

A positive definite kernel, *κ*: ℝ^*d *^× ℝ^*d *^→ ℝ, implicitly defines an embedding map

via the inner products in the feature space ℱ, i.e.

By evaluating spatial depth on the feature space ℱ, we obtain KSD which is(2)

With simple algebra, (2) can be rewritten as

where .

The value of KSD depends upon *κ *without knowing explicitly what the *ϕ *is. In ℝ^*d*^, one of the popular positive definite kernels is the Gaussian kernel *κ *(*x*, *y*) = exp(-||*x *- *y*||^2^/*σ*^2^), which can be interpreted as a *similarity *between *x *and *y*, hence it encodes a similarity measure. For a graph, we must consider what a good similarity measure will be, and how to construct an appropriate kernel matrix efficiently.

### Choice of graph kernels

Various kernels on graphs can be found in recent literature, for example [39-41], and [42]. Ando and Zhang [43] provide some theoretical insights into the role of normalization of the graph Laplacian matrix. We consider five Laplacian kernels, including *complement Laplacian kernel*, which is proposed here. Each kernel is described, followed by a comparison and discussion of computational issues of these kernels.

#### Laplacian kernel

For a given *G *= (*V*, *E*) and an associated weight matrix *W*, the Laplacian of the graph is *L *= *D *- *W *and the *normalized Laplacian kernel *is(3)

where *D *is a diagonal matrix with the diagonal entities being the degrees, i.e. *D*_*ii *_=  = Σ_*j*_*W*(*i*, *j*).

We confine our attention to a graph without self-loops, that is, *W*(*i*, *i*) = 0; *i *= 1,...,*n*, where *n *is the number of vertices. Then all the diagonal entities of ℒ are 1, and the off entry  if *v*_*i *_and *v*_*j *_are adjacent and 0 otherwise. By spectral graph theory [44], ℒ is positive definite symmetric and can be treated as a kernel matrix. We must determine how well it measures similarity between vertices of the graph. Let us calculate the distance between two vertices *v*_*i *_and *v*_*j *_in the feature space ℱ induced by ℒ.

From the above result, we can see that the distance between two adjacent vertices in the feature space is larger than that of two disconnected vertices. The mapping *ϕ *reverses the relationship between two vertices in the graph. In this sense, we can view the Laplacian kernel as a dissimilarity matrix. In other words, a vertex close to the center in the graph turns into a vertex far from the center in the feature space. Therefore, a smaller KSD value indicates a higher rank of the vertex in the graph when choosing the Laplacian as the kernel. It is interesting but not consistent with the usual kernels that describe the similarity between two vertices. Next we look at several alternatives to Laplacian kernel.

#### Laplacian of complement graph kernel

Considering the inverse of the Laplacian kernel, we may look for some kernels that can "turnaround" the Laplacian kernel. One natural way is to consider the Laplacian of the complement graph of the original graph. The complement of a graph *G*, denoted as , is the graph with the same vertex set but whose edge set consists of the edges not present in *G*. For simplicity, we only consider un-weighted graphs. Let *W *be the weight (adjacency) matrix and *D *be the degree matrix of graph *G *as above. The weight matrix  of the complement graph  can be expressed as

where *E *is the square matrix with all entries of 1 and *I *is the identity matrix. The degree matrix  of  is

Hence the Laplacian matrix of  is

Therefore we can choose  as the *complement Laplacian kernel*:(4)

There is no question that *nI *- *E *- *L *is symmetric and positive semi-definite. Notice that the Laplacian of the complement graph is defined in terms of negative Laplacian of the original graph. Hence it reverses the dissimilarity measure of *L*_*G*_. In other words, the Laplacian of the complement graph is a similarity matrix. Therefore, the larger KSD value with Laplacian of the complement as the kernel indicates the deeper the vertex is in the graph as we expect. This kernel is specially useful for dense graphs. The Laplacian of the complement of the graph may be a sparse matrix which leads to an efficient implementation of the KSD algorithm.

#### Diffusion Laplacian kernel

One could also consider other Laplacian based kernels that perform opposite operations of the Laplacian. One such meaningful alternative would be the diffusion kernel. The name "diffusion kernel" is due to the fact that it is a fundamental solution of the following *heat diffusion equation*:

In a way, the Laplacian is associated with the rate of diffusion of heat. For any *t *≥ 0, the diffusion kernel *H*_*t *_of *G *is

In particular, we take *H*_1 _as the *diffusion Laplacian kernel*:(5)

From Taylor expansion of exponential function, it is not difficult to show that **K**_*D *_is symmetric positive definite and all entries are non-negative.

**K**_*D *_can be computed from the spectral decomposition of ℒ, which is

where *U *is the matrix with columns being the eigenvectors and Λ is the diagonal matrix containing the eigenvalues of ℒ. Then

with .

Diffusion Laplacian kernel performs in an "opposite" way to the Laplacian kernel. Therefore like the Laplacian of the complement graph kernel, the larger KSD value using diffusion Laplacian kernel indicates the "central" vertex in the graph.

#### Pseudo-inverse Laplacian kernel

Similar to the diffusion Laplacian kernel, pseudo-inverse of the Laplacian is a kind of "opposite" operation. Due to the singularity of ℒ, the Penrose generalized inverse (pseudo inverse) is used for the kernel to represent the similarity between vertices of the graph.(6)

where Λ^- ^is a diagonal matrix with the (i, i) diagonal element being . For convenience, we define  = 0 if *λ*_*i *_= 0. Clearly, **K**_*P *_is also positive semi-definite, which means that it is indeed a valid kernel.

#### P-step random walk kernel

The last kernel we consider is called *p-step random walk kernel *with the form(7)

where *p *is a positive integer and *a *≥ 2. The name of the kernel is based on the fact that (*aI *- ℒ)^*p *^is up to scaling terms equivalent to a *p*-step random walk on the graph with random restarts. Since it involves negative ℒ in the form, it is a similarity kernel.

In particular, a *p*-step random walk kernel with *a *= 2 and *p *= 1, **K**_*R *_= 2*I *- ℒ, converts the off-diagonal dissimilarites in a Laplacian kernel to off-diagonal similarities. It is simple in form and is much more attractive for practical purposes.

### Ranking algorithm based on KSD for graphs

Given a graph *G *and a specified kernel, the following pseudocode describes the procedure to calculate the kernelized spatial depth values of all vertices.

#### Algorithm 1 KSD Algorithm

1 Get the Laplacian ℒ of the input graph G

2 Choose and compute the kernel matrix *K*

3 FOR (every vertex *m *in *G*)

4    FOR (every vertex *i *in *G*)

5       

6       IF *t *= 0

7          *α*_*i *_= 0

8       ELSE

9          *α*_*i *_= 1/*t*

10       END

11    END

12       FOR (every pair of vertices*i*, *j *in*G*)

13          *M*_*ij *_= *K*_*mm *_+ *K*_*ij *_- *K*_*mi *_- *K*_*mj*_

14       END

15    

16 END

17 OUTPUT*D*_*κ*_

From the above algorithm, the computation cost of KSD for all vertices depends on the sparseness of the kernel matrix. For a sparse kernel matrix, it is (*n*^2^), otherwise it is (*n*^3^). It is worthwhile to remark that the algorithm can be sped up by running it on multiple CPUs or computers even without the help of parallel programming techniques.

#### Comparison of kernels

**Figure 3:**
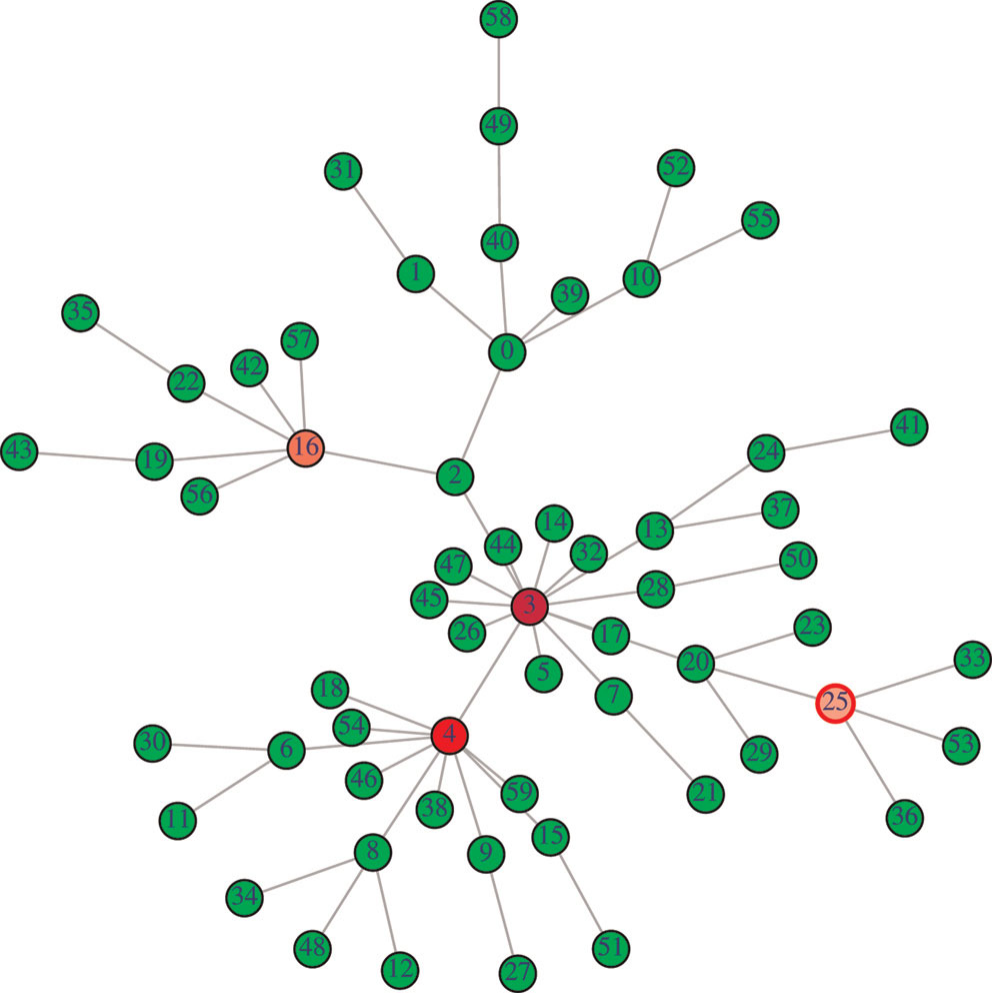
**Random graph for performance comparison of kernels**. For this random simulated sparse graph with 60 vertices, ranking by KSD using all five kernels and PageRank provide identical top 3 vertices: 3, 4, 16. Their top 10 lists also are very similar.

All five kernels are based on Laplacian which contains information of topological structure of the graph. From our simulated random graph data sets, they all perform well and similar. For example, Figure 3 is a random graph with 60 vertices. Ranking by KSD with all kernels and PageRank provide exactly the same top 3 vertices: 3, 4, 16. Their top 10 lists also are very similar. So our comparison of kernels focuses more on practical issues.

In the real world, most networks (graphs) such as the world wide web, biological networks including the gene-function bipartite graphs we will construct later, are sparse, which means that the associated weight matrices are sparse. Complement Laplacian kernel is not suitable because of its expensive computation cost (*n*^3^). Since the diffusion kernel and pseudo-inversion kernel require spectral decomposition of ℒ, which has (*n*^3^) complexity and also the resulting kernels usually are very dense, they are not attractive. The Laplacian kernel has some difficulty on interpretation, so we prefer to choose the p-step random walk kernel.

In our application work in the next section, we rank the importance of genes by KSD using the *p*-step random walk kernel with *a *= 2 and *p *= 1.

### Application to gene data

In our application, gene expression involving budding yeast (Saccharomyces cerevisiae) cells treated with DNA-reactive compounds cisplatin (CIS), methyl methanesulfonate (MMS), and bleomycin (BLE) to induce genotoxic stress will be compared with gene expression of Saccharomyces treated with DNA non-reactive ethanol (EtOH) and sodium chloride (NaCl) compounds to produce cytotoxic stress. Our goal is to identify a small number of biologically relevant genes capable of differentiating mechanisms of toxicity between the known genotoxic compounds from the cytotoxic compounds. In order to do so, we use the following basic methodology:

• Construct an unweighted gene-function bigraph based on GO with one partition representing genes and the other representing molecular function.

• Preprocess and combine data from the gene expression samples into one set per compound.

• For each compound, add weights to the bigraph using the gene expression data.

• Run the KSD algorithm on each bigraph to develop a gene expression profile of ranked genes for each compound.

• Compare the ranked gene sets.

Details of these steps are provided below.

### General construction of gene-function bigraph

In order to integrate biological information and gene expression data, one of gene ontologies – molecular function descriptions of genes are used. In the GO database, the ontologies are structured as rooted directed acyclic graphs (DAGs). The terms close to the root are more abstract than the terms far away from the root. We first extract the most specific functions associated with each gene to form the set of GO function terms. With one set of functions and the other set of genes, a bipartite graph is established. Consider Figure 3. Gene YGR098C is associated with the GO function term 0004197, which describes the cysteine-type endopeptidase activity. Genes YMR154C and YNL223W also have the same function. So in the bipartite graph, Gene YGR098C is more related to YMR154C and YNL223W than it is to YBL069W.

**Figure 4:**
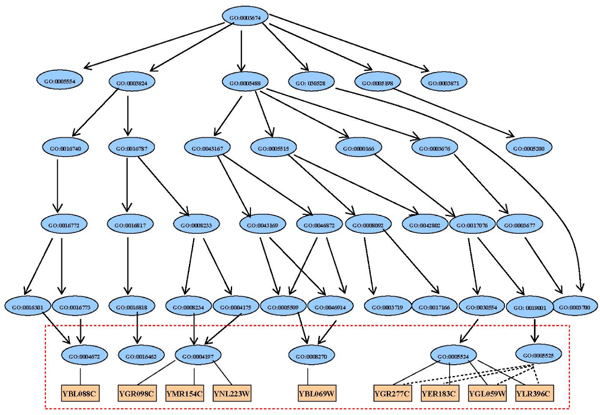
**Gene-function bigraph and DAG structure of ontology**. This plot demonstrates how to build the structure of gene-function bigraph. The orange rectangles at the bottom level represent genes. The blue ellipses and arrows above form a subgraph of the DAG in the GO database. Solid edges represent the association between genes and functions. Dashed lines are added edges that reflect the semantic similarity of function annotations. The graph inside the red dashed box is the gene-function bipartite graph.

To make the bigraph more informative, the "closeness" information between function terms are included into the bigraph. In this way, we not only use the low level (most specific level) descriptions but also the whole DAG structure of the ontology. For example, both GO term 0005524 (ATP binding) and 0005525 (GTP binding) belong to purine nucleotide binding (GO term 0017076). To represent this association, we add edges between GO:0005525 and the genes which are associated with GO:0005524. The added edges are represented as dash lines in Figure 4. The weights of the added edges will be the gene expression of the associated gene multiplied by a factor *r *∈ (0, 1), where *r *depends on the closeness of the two functions. We take *r *to be of the form *c*^*k*^, where *c *is a user-specified value between 0 and 1, and *k *is the number of up-trace levels in the GO DAG between the function and the lowest common ancestor shared by the two functions (a broader function description). The pseudocode of algorithm below states the procedure to construct the gene-function bigraph.

### Algorithm 2 Gene-Function bigraph Construction Algorithm

0 Input*c*, user specified parameter

1 Input gene data

2 Extract associate GO function terms*F*

3 Form weighted bigraph*G *= (*V*, *E*, *W*)

4 FOR each term *f*_*i *_in*F*

5    Obtain all ancestors*m *of*f*_*i *_and their generation levels*l*_*im*_

6 END

7 FOR every pair*i*, *j *in*F*

8    Find the nearest common ancestor*s*

9    *k *= max (*l*_*is*_, *l*_*js*_)

10    Add edges of*f*_*j *_and*g*_*t*_: (*g*_*t*_, *f*_*i*_) ∈ *E *with weights*W*_*ti *_× *c*^*k *^into*G*

11    Add edges of*f*_*i *_and*g*_*t*_: (*g*_*t*_, *f*_*j*_) ∈ *E *with weights*W*_*tj *_× *c*^*k *^into*G*

12 END

13 OUTPUT*G*

The construction of the gene-function bigraph combines gene expression profiles and topological similarity in a single framework. Khatri and Drăghici [45] summarized three ways to determine the abstraction level of annotation in their section 2.7. Our approach is a variation of their second method. The user may decide *k*, the bottom-up level, for annotations. The difference is that we treat the children terms unequally, similar to the weight strategy presented in [24].

Figure 3 demonstrates how to build the structure of gene-function bigraph. The yellow rectangles represent genes at the bottom level. The above blue ellipses and arrows form a subgraph of the DAG in the GO database. Solid edges represent the association between gene and function. Dashed lines are added edges that reflect the semantic similarity of function annotations. The graph inside the red dashed box is the gene-function bipartite graph.

### Preprocessing of gene expression data

**Figure 5:**
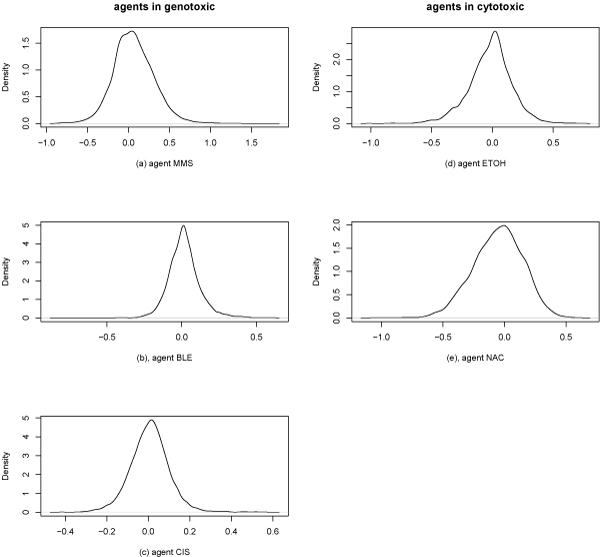
**Distribution of log2 gene expression difference wrt control agent for each treatment agent**. For each treatment agent, the probability density of log-2 expression differences is estimated and plotted. In general, yeast cells produce distinct gene expression responses to individual agents. However, some evidence shows the similarity of expression profiles between NaCl and EtOH, the two cytotoxic compounds. Their log-2 expression differences have a similar range and their density plots have similar long left tails compared with those with long right tails, CIS and MMS.

Our test data was obtained from the Gene Expression Omnibus (GEO), a database repository of high throughput gene expression data. We used the data set with access number GDS1299. The data were conducted by [46]. There are a total of 24 samples under 5 treatment agents with different dosages and control data. The series includes 11 control samples, 3 NaCl-treated samples, 2 EtOH-treated samples, 3 MMS-treated samples, 3 BLE-treated samples and 2 CIS-treated samples. We combine the data into one control set and 5 treatment sets by taking the averages of gene expression values within treatment group. For each gene, log base 2 of the ratio of treatment to control was used. The distribution of log-2 expression differences under each agent is given in Figure 5. The plot shows that, in general, yeast cells produce distinct gene expression responses to individual agents. However, some evidence shows the similarity of expression profiles between NaCl and EtOH, the two cytotoxic compounds. Their log-2 expression differences have a similar range and their density plots have similar long left tails compared with those with long right tails, CIS and MMS.

### Bigraphs for gene data under each treatment

In our application, we choose *c *= 1/5. Since *r *dramatically decreases on *k *for such choice of *c*, we truncate *r *to be zero for *k *> 1 to reduce computation memory and time. Under Algorithm 2, the bigraph under treatment MMS agent has total 5232 vertices including 4675 genes and 557 function terms. The number of edges are 22659. Hence the resulting bigraph is very sparse with sparsity 0.0017 comparing with 1 in the full graph (the graph with all pair edges). We use *p*-step random walk kernel to analyze the graph. Since we take log-2 expression differences with respect to the control agent, genes with positive log-2 expression difference are up-regulated and down-regulated genes have negative values. We are not able to directly assign weights of edges in the bigraph. We separate the bigraph into two subgraphs: one with all over-expressed genes and the other one with all under-expressed genes. For the subgraph containing "down-regulated" genes, the weights are assigned to be the absolute values of log-2 expression differences. Then we rank the important genes in those two graphs separately. It is reasonable to do so because we are interested in important induced genes and also repressed genes. All graph construction and algorithms are implemented using R and Bio-conductor.

### Validation of improvement using GO

Before we present the result on the genes that are able to potentially differentiate genotoxicity and cyto-toxicity, we would like to demonstrate that integrating GO will provide more reliable results than methods only using gene expression data. We consider the three NaCl samples individually, ranking differentially expressed genes in each sample and comparing the degree of overlap of the top 100 gene lists.

For the simplest fold-change method, which ranks genes by the ratio of expression level of a NaCl treated sample over the mean expression in the control group, there are seven common genes appearing in the top 100 of the three samples, and only three overlapping in the top 50. When t-statistics are used for ranking genes, there are no genes in the overlap of the top 50 genes from the three samples, and only five genes in the overlap of the top 100 genes. Moreover, only one gene is identified in each sample by both methods. The reasons for such a poor performance include the noise level and experimental variability of microarrays. Ranking each gene independently is also one of the attributed reasons. Incorporating gene expression profiles and biological knowledge can improve performance.

By integrating GO annotations, a gene-function bigraph is constructed with weights being fold-changes or t-statistics for each sample. The KSD ranking on fold-change weighted graph provides an overlap of 60 genes in the top 100 and 32 in the top 50. There are 45 common genes in all the top 100 and 24 in the top 50 if we rank the t-statistic weighted bigraph. Furthermore, 38 common genes are identified in every bigraph based on each sample using either a fold change or t-statistic. For other compounds, we obtained a similar result: a small overlap for methods on gene data alone, a relatively larger overlap for our approach on the GO derived graph. While our testing used GO function annotations, similar results are expected with the other two ontologies. It is noted that there is a complete overlap if only GO information is used. Gene-function bigraphs which combine gene data with GO enhance the experimental signal and capture the dependent structure of genes. Hence, ranking on bigraphs improves the results.

## Results

Applying the KSD ranking algorithm to the bigraphs constructed from gene expression responses to each agent, all induced and repressed genes were ordered separately according to their KSD values. The top 10 important genes are listed in Table 1. Our goal is to identify genes differentially modulated between the genotoxic and the cytotoxic agents. We first identify the genes that exhibit similar behavior in the groups. We then find those genes induced by one treatment group but repressed by the other treatment group.

**Table 1:** Top 10 induced (up) and repressed (down) genes for each agent.

	Genotoxic agents			Cytotoxic agents	
	
	MMS	Bleomycin	Cisplatin	EtOH	NaCI
Up	YJL088W	YMR090W	YJL088W	YPRWsigma4	YDR256C
	YNL241C	YPR160W	YMR090W	YJL088W	YLR343W
	YER161C	YGR180C	YGR180C	YML010W-A	YJR078W
	YJL101C	YNL202W	YER142C	YOL055C	YJL153C
	YDL142C	YGR256W	YNR019W	YLR067C	YNL275W
	YOR349W	YJR073C	YJL101C	YNL275W	YJL088W
	YDR019C	YOR100C	YJL026W	YNL036W	YER081W
	YDR001C	YDR018C	YKR076W	YER081W	YNL071W
	YBR045C	YJL026W	YLL060C	YLR237W	YBR221C
	YJL153C	YCR083W	YDR001C	YJL129C	YLR142W

Down	YNL327W	YNL327W	YNL327W	YJL178C	YDR435C
	YFL017C	YGL028C	YDR044W	YDL142C	YER009W
	YNL141W	YNR067C	YHR128W	YIL162W	YNL327W
	YOR095C	YGR006W	YHL028W	YCR021C	YGL143C
	YOL152W	YIL149C	YMR006C	YGR180C	YCR021C
	YOR356W	YPL276W	YOR095C	YGR277C	YDR071C
	YLR456W	YEL048C	YKL029C	YHR008C	YOL143C
	YHR128W	YER052C	YAR050W	YER023W	YOR121C
	YBR038W	YGL063W	YLL061W	YIR044C	YMR034C
	YGL143C	YOR095C	YNL148C	YML120C	YGL055W

We seek the genes that are on the top 50 list for all three treatments of genotoxic stress or for two cytotoxic agents. There are 17 genes common to DNA-reactive genotoxic compounds, of which 9 are induced and 7 are repressed. For two cytotoxic treatments, 5 over-expressed and 5 under-expressed genes are significant and common to EtOH and NaCl. See the Table 2. This clear overlap between genotoxic compounds, and similar overlap between the cytotoxic compounds gives added confidence that the profiles are stable. Gene EGT2 (ID: YNL327W) is an important gene that is down-regulated by all three genotoxic compounds. Gene RNR4 (ID:YGR180C) is the only gene that is up-regulated for genotoxic stress but down-regulated for cytotoxic stress. It encodes an essential small subunit of ribonucleotide reductase. It is known to be induced by DNA replication and DNA damage checkpoint pathways via localization of the small subunits in response to genotoxic stress.

**Table 2:** Genes with similar responses under genotoxic or cytotoxic stress

Genotoxicity		Cytotoxicity	
Induced (Up)	Repressed (Down)	Induced (Up)	Repressed (Down)

YJL088W	YNL327W	YJL088W	YCR021C
YDR001C	YOR095C	YNL275W	YGR180C
YJL178C	YGR036C	YER081W	YBR054W
YMR090W	YHL028W	YOR298W	YNR001C
YOR100C	YGL028C	YLR343W	YDR408C
YLR178C	YIL149C		
YGL156W	YGL063W		
YJR073C	YGR006W		
YGR180C			

Responses of the genes in this table are similar for genotoxic or cytotoxic stress. They are the common genes that appear in the top 50 induced or repressed list for DNA-reactive treatments or non-reactive treatments.

We enlarge the search of differentially regulated genes between the two groups to the top 100 genes. Eight other genes are capable of discriminating between genotoxic and cytotoxic agents. They behave similarly within group but totally different between groups. Genes over-expressed for genotoxic treatments but down-regulated for cytotoxic agents include TFS1, NTH1, ATG27 and un-characterized YMR090W. TFS1 is a Carboxy peptidase Y inhibitor, which is targeted to vacuolar membranes during stationary phase and involved in protein kinase A signaling pathway. NTH1 is required for thermotolerance and may mediate resistance to other cellular stresses. Type I membrane protein, ATG27, is involved in autophagy and the cytoplasm-to-vacuole targeting pathway. For gene YMR090W with unknown function, we should treat it with caution. GO term 0003674 is manually created for unknown molecular functions. Because our method utilizes the GO DAG structure, the identification of YMR090W may be caused by 0003674 (unknown function) but not by significant changes of mRNA levels. Further study about this gene is worthwhile.

Four genes PUS2, CAX4, WSC4 and MLP2 are induced for cytotoxic stress but repressed for genotoxic stress. PUS2 protein is a mitochondrial tRNA, associated with pseudouridine synthase activity targeted to mitochondria, specifically dedicated to mitochondrial tRNA modification. Response to decreased yeast viability and slow growth caused by cytotoxic stress, CAX4 is induced to increase the level of N-linked glycosylation. WSC4 is an ER membrane protein involved in the translocation of soluble secretory proteins and insertion of membrane proteins into the ER, which plays an important role in the stress response. MLP2, a Myosin-like protein associated with the nuclear envelope, connects the nuclear pore complex with the nuclear interior and is involved in the Tel1p pathway that controls telomere length.

A summary of the results is listed in Table 3 which provides a gene profile that can potentially distinguish between genotoxic and cytotoxic stresses.

**Table 3:** Significant important genes distinguishing genotoxicity and cytotoxicity

Gene ID	Gene Name	Description
YGR180C*	RNR4	Ribonucleotide-diphosphate reductase (RNR)
YLR178C*	TFS1	Carboxypeptidase Y inhibitor
YDR001C*	NTH1	Neutral trehalase, degrades trehalose
YJL178C*	ATG27	Type I membrane protein
YMR090W*		unkown function
YGL063W	PUS2	Mitochondrial tRNA:pseudouridine synthase
YGR036C	CAX4	Dolichyl pyrophosphate (Dol-P-P) phosphatase
YHL028W	WSC4	ER membrane protein
YIL149C	MLP2	Myosin-like protein associated with the nuclear envelope

Genes selected in this table are capable of discriminating between genotoxic and cytotoxic agents. They behave similarly within group but totally different between groups. Genes with * are over-expressed for genotoxic treatments but down-regulated for cytotoxic components. Genes without * are induced for cytotoxic stress but repressed for genotoxic stress.

Genes with * are over-expressed under treatment of genotoxic stress but under-regulated for cytotoxic stress. Genes without * are induced for cytotoxic but repressed for genotoxic compounds.

### Comparison with PageRank

We also use PageRank to analyze each weighted bigraph under each treatment. It yields very similar results as our KSD. Considering up-regulated genes for MMS, 85 out of the top 100 ranked genes by PageRank coincide with the top 100 by KSD. For down-expressed genes in the MMS treatment, there are 77 common genes appearing in both top 100 lists by PageRank and KSD. The other compounds have a similar overlap in top 100 lists. PageRank and KSD produce similar ranking lists for gene data, so why do we need KSD?

There are two major advantages of KSD over PageRank. First, PageRank needs a damping parameter to be specified. From some empirical studies, the parameter being 0.85 (the default value in R) seems to work well on the balance between the convergence rate and stability in many applications. But there are some circumstances where 0.85 may be far from the "optimal" value. The choice of the damping parameter is a concern for PageRank and hence for GeneRank also. This is however not an issue for KSD if we use Laplacian, complement Laplacian or Psedo-inverse kernels. Second, since spatial depth is a robust measure for centrality, we expect that KSD will inherit this nice property and obtain a more robust ranking result. To demonstrate the robustness, we design the following experiment to compare the sensitivity of our approach and PageRank against incorrect annotations on the artificial data.

**Figure 6:**
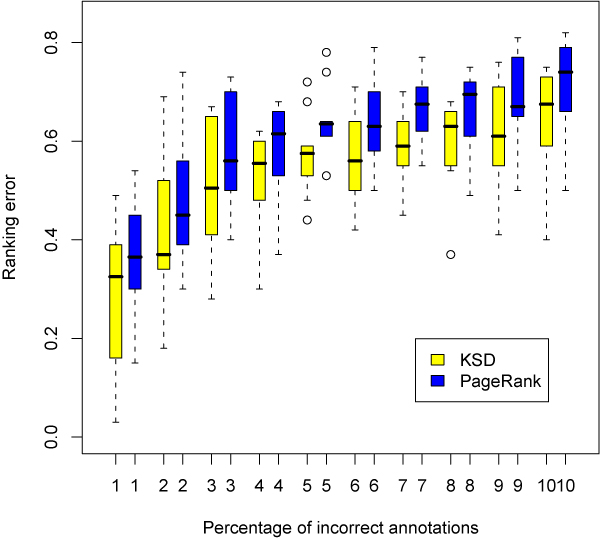
**Boxplots of robustness comparison of KSD and PageRank**. We randomly generate contaminated graphs with a certain percentage of incorrect annotations. Ranking error is defined as the difference percentage of the top 100 ranking list on the contaminated graph and the original graph. The boxplots are based on 10 random replications. KSD has a lower ranking error than PageRank. It shows that KSD is more robust than PageRank against incorrect annotations.

Suppose that all annotations are correct in the original bigraphs. We generate incorrect annotations by removing and adding some edges. For the weighted up-regulated gene-function bigraph under MMS, we randomly select a certain percentage of genes. For each selected gene, we randomly delete one of its correct annotations (one of edges between the selected gene and its corresponding functions) and add an incorrect annotation. The resulting bigraph contains contaminated data with incorrect annotations. We compare the ranking list on the contaminated graph and the one on the original graph by KSD and PageRank. We define a ranking error as the percentage difference between the top 100 ranking lists. Figure 6 provides the boxplots of ranking error versus the contamination percentage for KSD and PageRank where each boxplot is based on 10 random repeats. KSD has lower ranking errors than PageRank. On average, the ranking error of KSD is 6% lower than PageRank, which means that on the contaminated data, KSD can identify 6 more important genes than PageRank. This shows that KSD is more robust than PageRank. Given that microarray data is often "noisy", robustness is an important requirement for any method used with microarray data.

## Conclusion

The gene-function bigraph integrates molecular function annotations with gene expression data. The general relevance of genes is described in the graph (through a common function). Weights of the graph are assigned to be gene response expressions. The resulting bigraph includes more biological information than the gene data alone. Consequently, ranking on the bigraph may provide more biologically significant genes than ranking procedures based only on gene data. Also, we propose a new ranking algorithm for graphs based on the KSD measure. KSD balances the local and global topological structure of the graph, hence it provides a good and meaningful ordering of vertices of the graph. Experimental results on artificial data show that KSD is more robust than the well-known PageRank against incorrect annotations. The proposed method provides an exploratory framework for gene data analysis.

## Competing interests

The authors declare that they have no competing interests.

## Authors' contributions

XD and YC contributed to the theoretical development of the ranking algorithm. DW contributed to the bigraph formulation of GO data set. CG and DW contributed to the experiment and development of the computer code. CG and XD contributed to the draft. All authors read and approved the final manuscript.
